# Optimizing cardiac amyloidosis assessment: utility of 1-h and 3-h ^99m^Tc-PYP imaging

**DOI:** 10.1186/s40001-023-01629-y

**Published:** 2024-01-06

**Authors:** Aiganym Imakhanova, Reiko Ideguchi, Hiroaki Kawano, Koji Maemura, Takashi Kudo

**Affiliations:** 1 Department of Radioisotope Medicine, Nagasaki University Graduate School of Biomedical Sciences, Nagasaki University, Sakamoto 1-12-4, Nagasaki, 852-8523 Japan; 2 Department of Radioisotope Medicine, Atomic Bomb Disease Institute, Nagasaki University, Nagasaki, Japan; 3 Department of Cardiovascular Medicine, Nagasaki University Graduate School of Biomedical Sciences, Nagasaki University, Nagasaki, Japan

**Keywords:** ATTR transthyretin cardiomyopathy, Nuclear imaging, ^99m^Tc-PYP, Incubation time

## Abstract

**Background:**

Transthyretin amyloid cardiomyopathy (ATTR-CM), characterized by the extracellular deposition of an insoluble amyloid protein in the heart, is one of the main causes of heart failure in elderly patients. In this study, our primary objective was to explore the diverse applications and temporal significance of 1-h and 3-h imaging using ^99m^Tc-PYP in the context of ATTR-CM. Additionally, we compared tracer kinetics in the heart and bone to comprehensively assess the diagnostic advantages and time-related considerations associated with these two incubation periods.

**Methods:**

Twenty-seven patients at Nagasaki University Hospital who underwent ^99m^Tc-PYP planar, and SPECT cardiac imaging were classified into two groups (ATTR-CM-positive and -negative groups) based on the American Heart Association statement. Cardiac retention was assessed with both a semiquantitative visual score and a quantitative analysis. To assess bone accumulation, a ROI with an equal volume was drawn on the sternum and calculated as the bone-to-contralateral ratio (B/CL). We also evaluated correlation between heart-to-contralateral lung (H/CL) ratio and left ventricular wall thickness.

**Results:**

Among patients who underwent ^99m^Tc-PYP imaging, the H/CL ratio was significantly higher at 1 h than at 3 h regardless of the group (from 2.20 ± 0.36 to 1.99 ± 0.35, *p* < 0.01 in the positive group and from 1.35 ± 0.12 to 1.19 ± 0.21, *p* = 0.01 in the negative group). The gap of H/CL between highest H/CL of negative case and lowest H/CL of positive case was narrower in 3 h. On the other hand, correlation between H/CL and left ventricular posterior wall thickness tends to be clearer in 3 h (*p* = 0.12, *r* = 0.30 for 1 h, *p* = 0.04, *r* = 0.39 at 3 h).

**Conclusion:**

Our study suggests that both 1-h and 3-h incubation times for ^99m^Tc-PYP imaging have different benefits for ATTR cardiac amyloidosis. A one-hour incubation may be preferable for differential diagnostic purposes, while a three-hour incubation may provide greater utility in evaluating disease severity.

## Introduction

Transthyretin amyloid cardiomyopathy (ATTR-CM) is caused by a mutation in the gene encoding transthyretin, a small molecule mainly produced by the liver [1]. Cardiac involvement is considered to be the leading cause of morbidity and mortality in systemic amyloidosis [2]. An epidemiological multicenter study conducted in Finland [3] showed that the prevalence of ATTR in an autopsied elderly population was high at 25%. Another study performed in Spain indicated that approximately 13% of patients with heart failure with a preserved ejection fraction (EF) and older than 60 years had ATTR cardiac amyloidosis [4]. Based on these findings, cardiac amyloidosis, particularly ATTR cardiac amyloidosis, appears to be severely underdiagnosed.

^99m^Tc-PYP is an avid bone radiotracer that has been clinically used in myocardial infarct imaging for more than 40 years. Early single-center studies [5] used modern amyloid subtypes and demonstrated the excellent diagnostic efficacy of scintigraphy ^99m^Tc-PYP for ATTR-CM. Since ^99m^Tc-PYP and other imaging modalities using bone tracers are reliable, recent guidelines from the American Heart Association (AHA) [6] and European Society of Cardiology [7] both issued statements on the diagnosis of cardiac amyloidosis that limited the requirement for biopsy to cases with equivocal/questionable findings on bone tracer images.

Although the effectiveness of ^99m^Tc-PYP has been demonstrated, the question of standardizing the diagnostic protocol remains open. ^99m^Tc-PYP imaging has been used in the imaging of bone and acute myocardial infarction, for which the recommended standard incubation time is 3 h. The cardiac ^99m^Tc-PYP protocol continues to evolve, with centers performing planar and SPECT imaging at 1 or 3 h. However, a standardized incubation time has not yet been established. In studies based on previously published amyloid imaging data, Columbia University [8] and Boston University [9] performed planar imaging of the heart after an incubation time of 1 h, and the H/CL ratio to indicate ATTR cardiac amyloidosis was assumed to be 1.5 or higher. At the same time, the Mayo Clinic used a 3-h incubation, and the threshold value for the H/CL ratio was 1.3 or higher [8]. In their study [9] imaging with different incubation times was mixed and analyzed as a combined dataset without a detailed explanation. Furthermore, a Practice Points document [10] from the American Society of Nuclear Cardiology (ASNC) and a multi-society consensus document described obtaining planar images 1 and 3 h after a radiotracer injection, in addition to single-photon emission computed tomography with computed tomography (SPECT/CT) to confirm that visualized ^99m^Tc-PYP localized to the myocardium and was not just in the blood pool.

To resolve this confusion, we considered the necessity of standardizing the incubation time for imaging ATTR cardiac amyloidosis using ^99m^Tc-PYP. To achieve this, we performed comparisons of quantitative parameters measured with planar imaging between images acquired 1 and 3 h after the injection of a tracer.

## Materials and methods

### Subjects

Data collected from 28 patients suspected of having cardiac amyloidosis based on clinical findings and who underwent ^99m^Tc-PYP nuclear scintigraphy at Nagasaki University Hospital between October 2017 and February 2020 were retrospectively analyzed. This is a retrospective study using routine clinical examination. Ethical approval was granted by the Ethics Committee of Nagasaki University Hospital (Approve No.: 20111631), with a waiver of written informed consent in view of the retrospective nature of the study. All procedures conducted in the studies involving human participants were in accordance with the 1964 Helsinki Declaration and its later amendments or comparable ethical standards.

### Image acquisition

Patients were intravenously injected with 700–900 MBq of ^99m^Tc-PYP and planar images in the anterior and lateral projections were obtained 1 and 3 h later. SPECT was performed after the acquisition of planar images at 3 h. The data from SPECT images are not used in the present study, except for the Perugini grading of the cases.

Planar imaging and SPECT/CT were conducted using a Symbia T SPECT/CT camera (Siemens Germany). Regarding planar images, data were acquired using a 128 × 128 matrix, low energy high-resolution collimator, and 15% energy window. In SPECT/CT, the same energy window with a 64 × 64 matrix and 360° rotation were used with CT attenuation corrections.

### Image interpretation

Images were analyzed using two methods. The semiquantitative visual scoring of cardiac retention applying the Perugini score [11] was performed using planar and SPECT/CT images. Grades 2 and 3 were considered to be positive findings according to the AHA statement. In the quantitative analysis [12], a circular region of interest (ROI) was drawn over the heart on anterior images in the standard manner, copied, and mirrored over the contralateral chest. The fraction of mean counts in the heart ROI-to-contralateral chest ROI was calculated as the heart-to-contralateral (H/CL) ratio. This calculation was performed on images acquired at 1 and 3 h, followed by a comparison of counting. To assess bone accumulation, an equal volume with an irregular ROI was drawn over the sternum on anterior planar images. The proportion of mean values in the ROI of the sternum in relation to the contralateral area of the chest was calculated as the bone-to-contralateral (B/CL) ratio. The B/CL ratio of images acquired at 1 and 3 h was compared. OsiriX MD 11.0 software (Pixmeo, Switzerland) was used for image interpretation and analysis.

**Fig.1:**
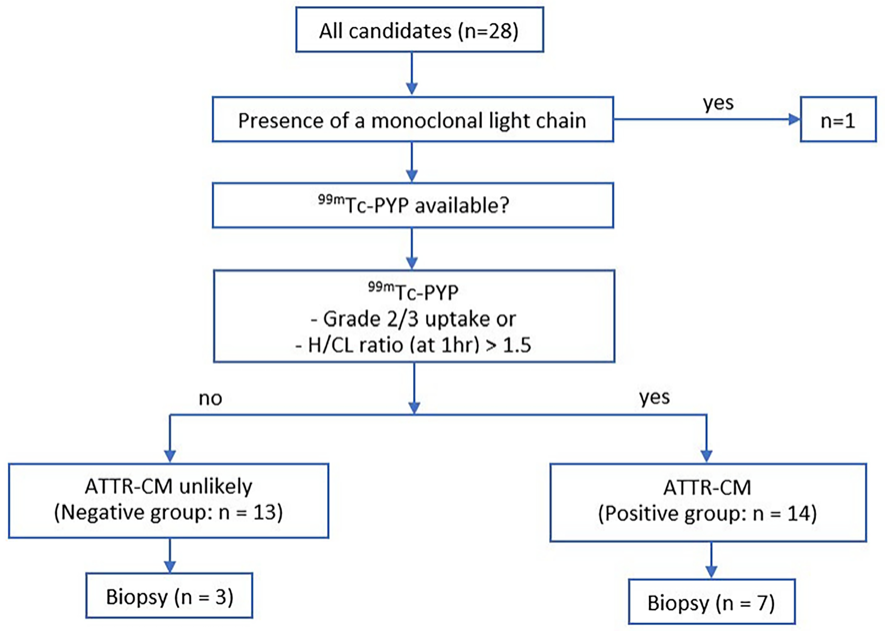
Final diagnostic tree. Patients are diagnosed with ATTR cardiac amyloidosis (in this tree described as ATTR-CM: ATTR cardiomyopathy) according to the AHA statement. Due to risks associated with myocardial biopsy, it was only performed on a few patients. *N*: number; ^99m^Tc-PYP: ^99m^Technetium-pyrophosphate; H/CL ratio: a heart-to-contralateral lung (H/CL) ratio; hr: hour; ATTR-CM: Transthyretin amyloid cardiomyopathy

We chose to employ the diagnostic algorithm outlined in the American Heart Association (AHA) statement as the gold standard for diagnosing amyloid cardiomyopathy, subsequently creating the diagnostic flowchart depicted in Fig. 1 based on this framework [6]. Briefly, the possibility of other cardiac diseases, such as hypertrophic cardiomyopathy and constrictive pericarditis, was investigated. Patients demonstrating evidence of monoclonal light chains were excluded from the analysis. The remaining patients were diagnosed with cardiac amyloidosis (positive group) when ^99m^Tc-PYP imaging indicated a H/CL ratio (measured on planar image obtained after 1-h incubation time, according to the ASNC guidelines) > 1.5 or a visual uptake (Perugini grade) equal to 2 or 3. Visual uptake grading was conducted by two interpreters, TK, a nuclear cardiologist with 30 years of experience, and AI, a researcher with 4 years of expertise in cardiac amyloidosis, using both planar and SPECT images, based on their mutual agreement. Patients not meeting these criteria were categorized into the negative group. These two groups were then compared. In our study cohort of 27 patients, grouping by visual assessment (Perugini score) and quantitative methods (H/CL ratio at 1 h) showed complete agreement, comprising 13 negative and 14 positive cases by both methods. No discordant cases were found, indicating a strong concordance between visual and quantitative methods for the diagnosis of ATTR cardiac amyloidosis in our cohort.

Biopsy was performed in cases in which ^99m^Tc-PYP results were negative, but the possibility of ATTR cardiac amyloidosis was still negligible based on clinical symptoms/findings, or in cases in which differentiation between mutant and wild-type ATTR cardiac amyloidosis was required for therapeutic reasons. There are no cases where biopsy changed diagnosis using ^99m^Tc-PYP imaging.

### Statistical analysis

Statistical analyses were performed using JMP Pro15 software. Demographic, laboratory, and imaging data were collected and analyzed with descriptive statistics using means ± standard deviations for continuous variables and relative percentages for categorical variables. P-values were calculated using the Shapiro–Wilk test to examine the normality of each variable. Fisher’s exact test was performed for categorical variables, an independent-sample *t*-test for normally distributed continuous variables, and an independent-sample Mann–Whitney U test for non-normally distributed continuous variables. A non-paired *t*-test was used to assess differences in continuous variables between study subgroups. The Pearson correlation coefficient analysis was also performed to assess the relationship between H/CL values and other variables. *p*-values < 0.05 were considered to be significant.

## Results

Twenty-eight patients who underwent ^99m^Tc-PYP nuclear scintigraphy to evaluate cardiac amyloidosis at Nagasaki University Hospital between October 2017 and February 2020 were enrolled in the present study. One patient with AL amyloidosis was excluded from the analysis (Fig. 1). The remaining patients were classified into the ATTR-CM group (positive group) and non-ATTR-CM group (negative group). The demographic, clinical, and echocardiographic features of subjects are shown in Table 1. Among the 27 patients examined, 21 (77.8%) were older male adults with a mean age of 75 years. Seventeen (63%) patients in the study cohort had New York Heart Association (NYHA) Class III/IV heart failure with an average EF of 44.3 ± 15.6%. Troponin T and calcium levels as well as the H/CL ratio were higher in the positive group. Interventricular septum thickness and LV posterior wall thickness were significantly greater in the positive group than in the negative group. Eight patients in the examined population had a history of angina pectoris, and two had angina at the time of imaging.

**Table 1:** Baselines mean clinical, laboratory, and echocardiographic characteristics

Category	Overall cohort (*n* = 27)	Negative group (*n* = 13)	Positive group (*n* = 14)	*p*
Clinical				
Age (yrs)	75 ± 11	71.3 ± 13.6	79.2 ± 6.1	0.05
Male sex (%)	21 (77.8%)	8 (61.5%)	13 (92.9%)	0.07
BMI, kg/m^2^	22.9 (15.9 – 34.8)	22.7 (15.9–34.9)	23 (19.5–26.5)	0.816
NYHA class III, IV	17 (63%)	7 (53.83%)	10 (71.4%)	0.44
History of angina	8 (29.6%)	3 (23.1%)	5 (35.7%)	0.678
Present angina	2 (7.4%)	2 (15.4%)	–	0.222
Coronary artery disease	5 (18.5%)	2 (15.4%)	3 (21.4%)	1.0
Hypertension	20 (74.1%)	9 (69.2%)	11 (78.6%)	0.678
Hyperlipidemia	7 (25.9%)	2 (15.4%)	5 (35.7%)	0.385
Laboratory				
Troponin T (ng/mL)	0.070 (0.016–0.153)	0.031 (0.016–0.098)	0.094 (0.034–0.153)	0.06
Creatinine (mg/dL)	1.2 ± 0.7	1.3 ± 1	1.2 ± 0.3	0.590
Calcium (mg/dL)	9.12 ± 0.57	9.08 ± 0.39	9.16 ± 0.75	0.754
NT-proBNP (pg/mL)	7131 (182–25,909)	4689 (182–25,909)	9573 (577–17,307)	0.219
GFR (mL/min)	46.2 ± 15.4	48.3 ± 21.4	46.1 ± 10.4	0.308
Echocardiographic				
EF (%)	44.3 ± 15.6	47.6 ± 16.4	41.2 ± 14.6	0.298
EDV (mL)	97.36 ± 38.2	112 ± 45.3	83.2 ± 26.2	0.056
ESV (mL)	50.3 ± 27.2	54.7 ± 36.2	41.2 ± 20	0.207
LA volume (mL)	91.87 ± 38.1	104.1 ± 47.6	78.4 ± 17.9	0.108
Interventricular septum thickness (mm)	15.2 ± 2.7	14.1 ± 2.3	16.2 ± 2.8	0.044
LV posterior wall thickness (mm)	14.1 ± 3	13.1 ± 2.1	15.1 ± 3.6	0.105

Continuous data are expressed as the mean ± standard deviation and categorical data as percentages

*N*: number; *p*: *p*-value; yrs: years; BMI: body mass index; kg/m^2^: kilograms per square meter; NYHA: New York Heart Association; ng/mL: nanograms per milliliter; mg/dL: milligrams per deciliter; NT-proBNP: N-terminal prohormone of brain natriuretic peptide; pg/mL: picograms per milliliter; GFR: glomerular filtration rate; mL/min: milliliters per minute; EF: ejection fraction; EDV: end-diastolic volume; mL: milliliter; ESV: end-systolic volume; LA: left atrial; LV: left ventricular; mm: millimeter

**Fig. 2:**
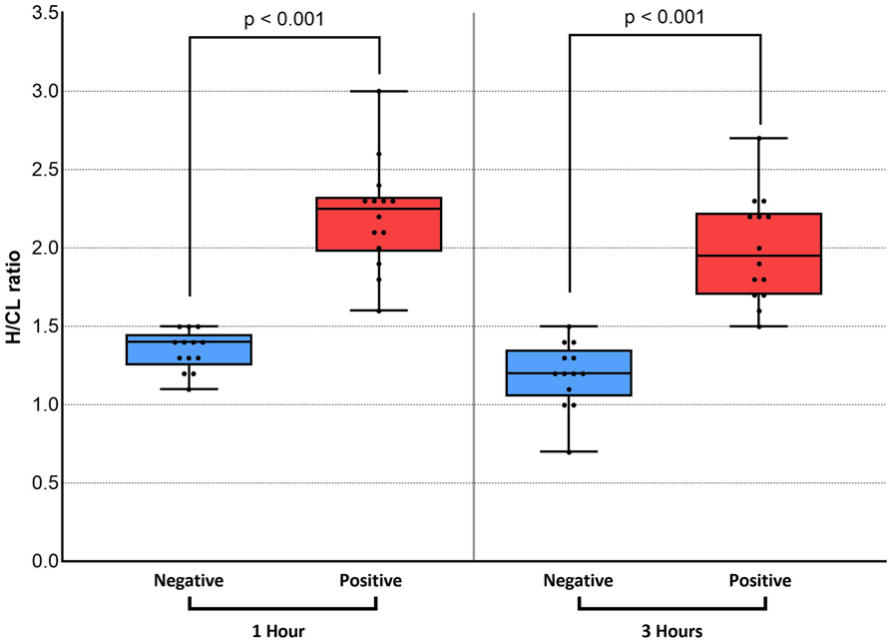
Comparison of data collected at 1 and 3 h in visually positive vs negative groups. Data on the H/CL ratio measured at 1 and 3 h are shown. At 1 h, there was no patient in the negative group with a higher H/CL value than the minimum in the positive group. At 3 h, one patient in the negative group showed a higher value than the minimum in the positive group. This patient was histologically proven to not have ATTR cardiac amyloidosis. H/CL ratio: a heart-to-contralateral lung ratio

**Fig. 3:**
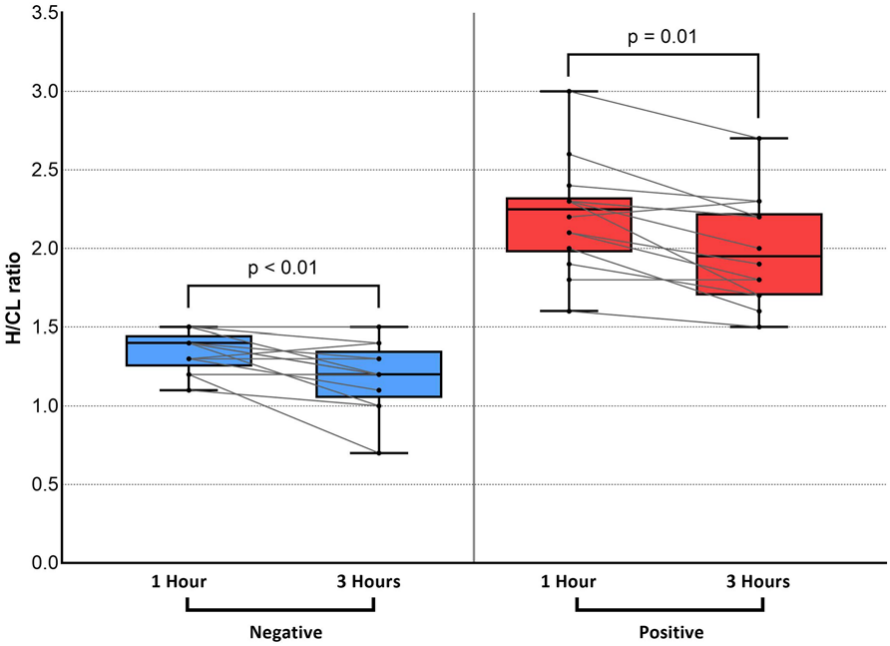
H/CL ratio change between 1 and 3 h in negative and positive groups. The left panel shows the negative group, and the right panel shows the positive group. The majority of patients regardless of the group classification showed a decline in the H/CL ratio at 3 h. Only a single patient in each group showed a change in the opposite direction. H/CL ratio: a heart-to-contralateral lung ratio

**Fig. 4:**
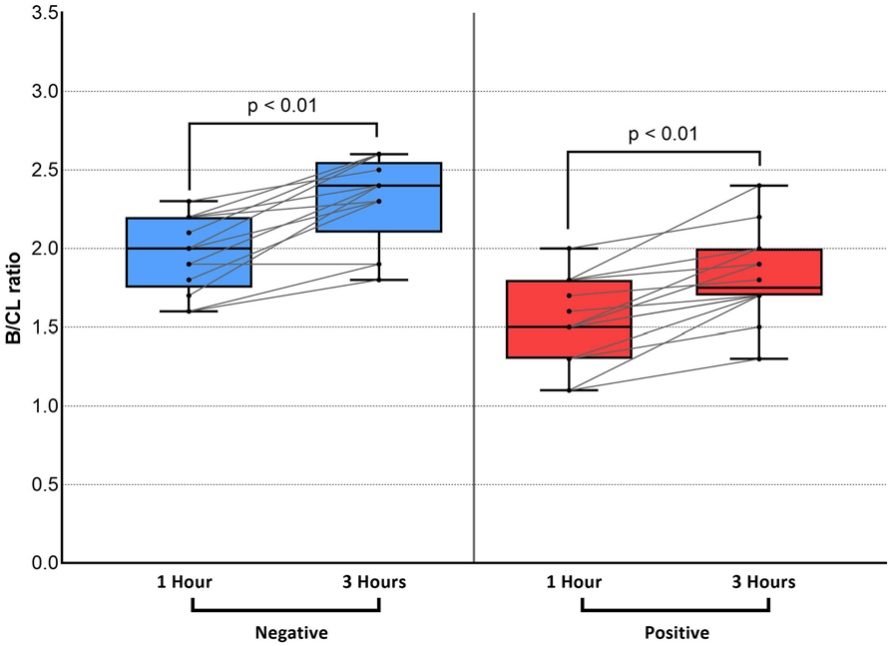
B/CL ratio change between 1 and 3 h in negative and positive groups. No patient in either group showed a decline in the B/CL ratio at 3 h. B/CL ratio: bone-to-contralateral lung ratio

**Fig. 5:**
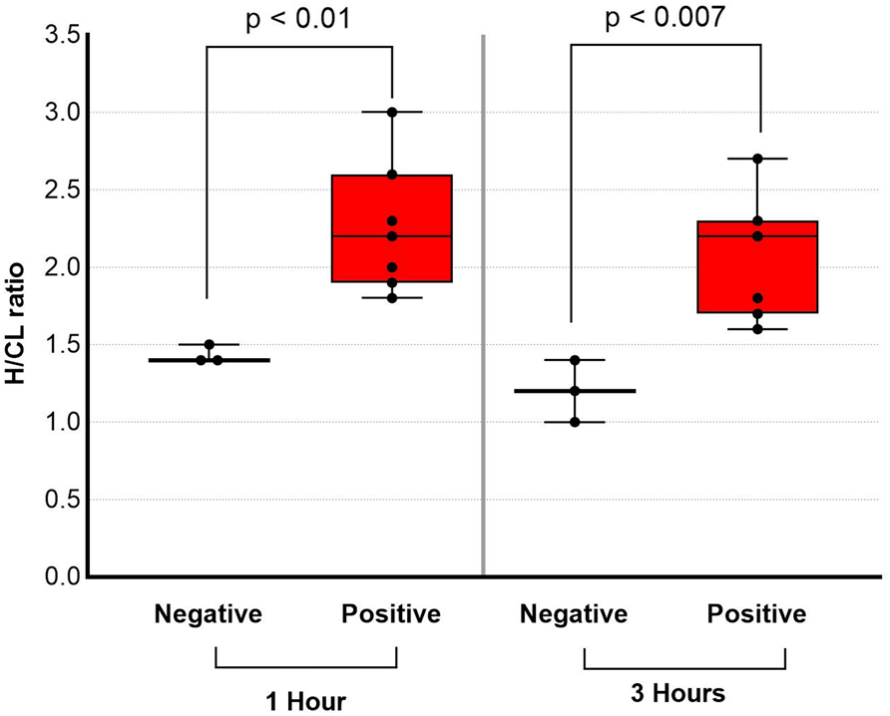
H/CL ratio of histologically proven 10 cases. Note the highest H/CL ratio of histologically proven negative case in 3 h is 1.4. H/CL ratio: a heart-to-contralateral lung ratio

**Fig. 6:**
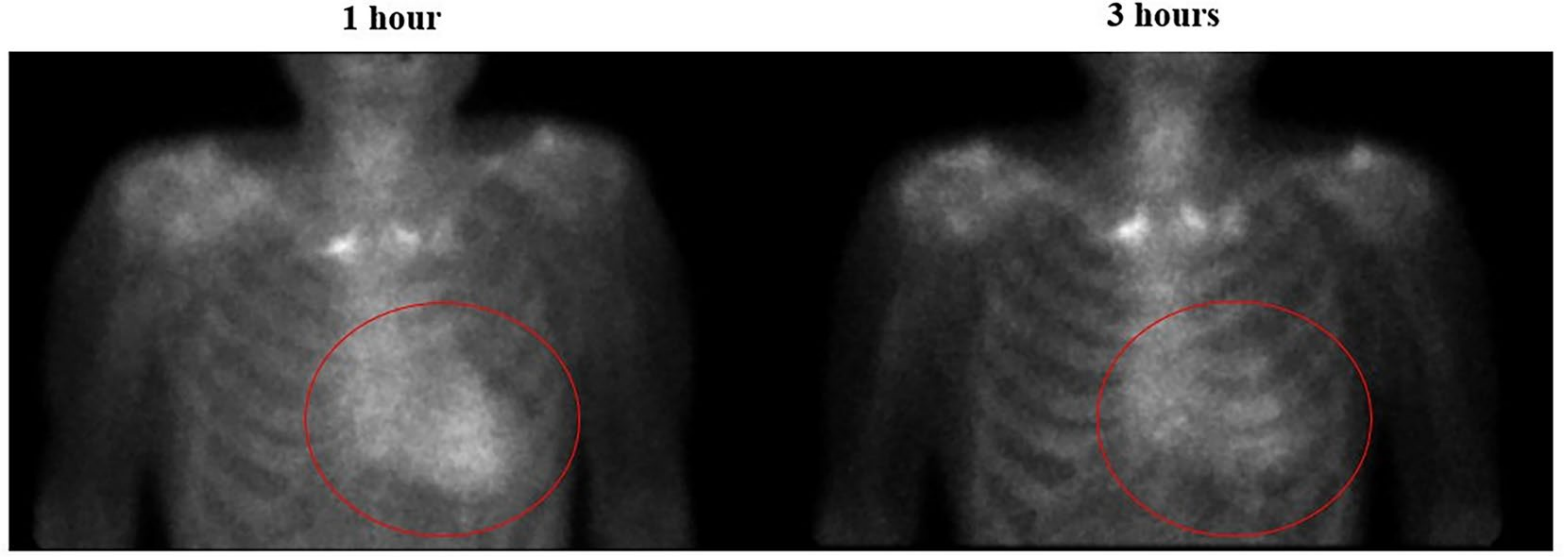
Planar anterior images of a patient 1 and 3 h after the injection of ^99m^Tc-PYP. Planar anterior images of 84 years old man in 1-h and 3-h after injection of PYP. Images show a strong reduction in the uptake of ^99m^Tc-PYP. The visual scoring of cardiac retention at 1 h was grade 3 and the H/CL ratio was 2.3, while that at 3 h was 1 and the H/CL ratio was 1.8. Please take note that within the figure, the red circle delineates the visual representation of reduced heart uptake

The results of comparisons of the H/CL ratio in the positive and negative groups at 1 and 3 h are summarized in Fig. 2. Data obtained at 1 and 3 h significantly differed between the two groups. However, the overlap between the two groups was smaller for data collected at 1 h. Figure 3 shows changes in the H/CL ratio in each patient between 1 and 3 h. Among patients who underwent ^99m^Tc-PYP imaging, the H/CL ratio was significantly higher at 1 h than at 3 h in both of positive and negative groups (2.20 ± 0.36 vs 1.99 ± 0.35, *p* < 0.01 for positive, 1.35 ± 0.12 vs, 1.19 ± 0.21, *p* = 0.01 for negative group). Regardless of the group classification, the majority of patients had a lower H/CL ratio at 3 h. Only a single patient in each group showed a change in the opposite direction. Figure 4 shows changes in bone uptake, expressed as the B/CL ratio, at 1 and 3 h in each patient. In contrast to the H/CL ratio, the majority of patients, regardless of the group classification, had a higher B/CL ratio at 3 h (1.96 ± 0.24 to 2.31 ± 0.28 for negative, *p* < 0.01, and 1.54 ± 0.27 to 1.82 ± 0.28, *p* < 0.01 for positive group). Figure 5 compares H/CL1h and H/CL3h ratios in 10 cases where we were able to obtain cardiac biopsies, confirming the diagnosis. Due to the challenges of performing biopsies in cardiac amyloidosis cases, our dataset is limited. The 3-h dataset shows a narrower gap between the highest-ranked negative and lowest-ranked positive cases than that in 1-h dataset. Figure 6 shows a typical patient showing a strong decline in the myocardial uptake of PYP. In this patient, the H/CL ratio (2.3 > 1.8) and visual uptake markedly decreased and reached a score = 1 at 3 h.

**Fig. 7:**
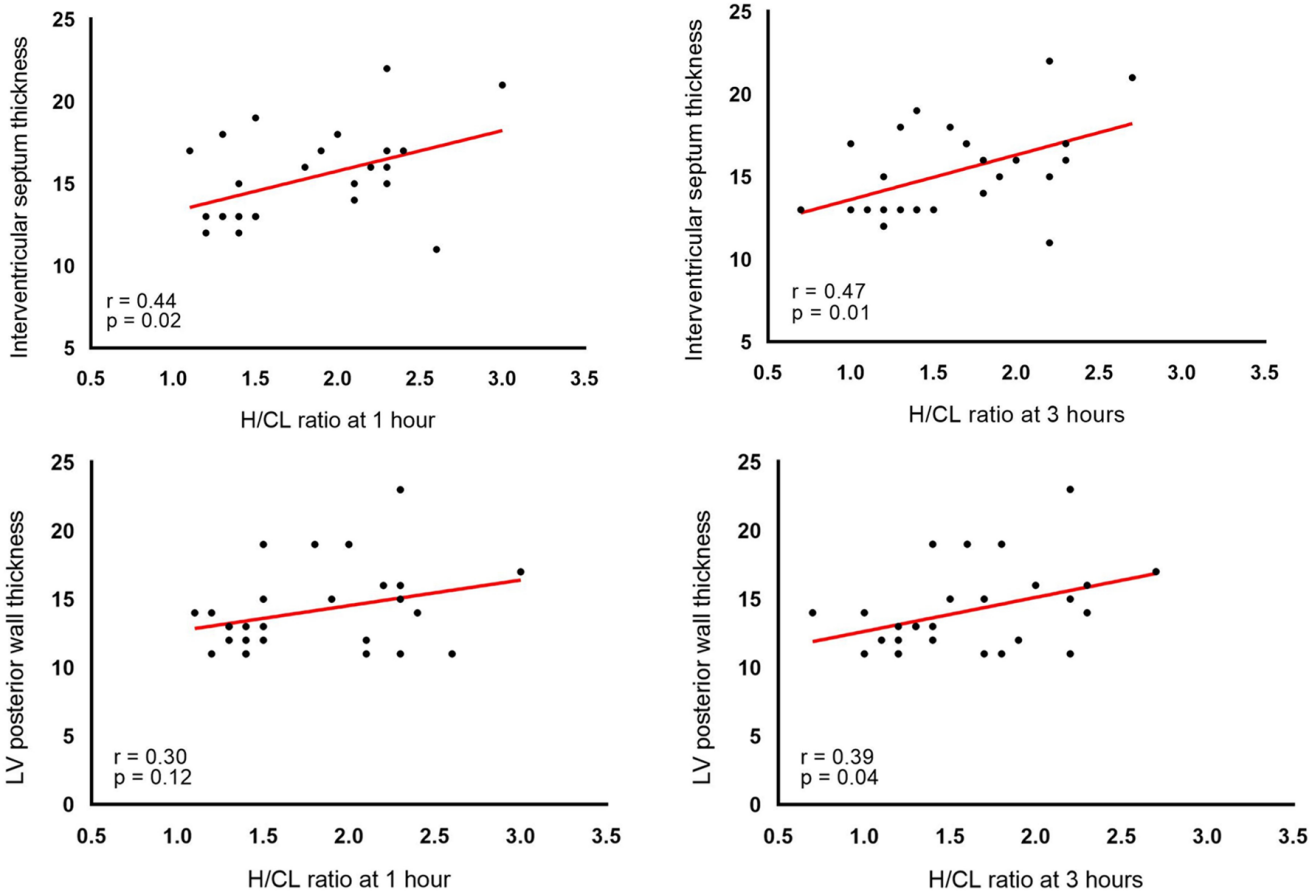
Correlation between the H/CL ratio (1 and 3 h) and the thickness of interventricular septum and posterior wall thickness. H/CL ratio: a heart-to-contralateral lung ratio

We also examined the relationships between cardiac function parameters and the H/CL ratio (Table 2 and Fig. 7). Correlation analysis showed that both H/CL measured at 3 h and 1 h showed significant correlation with intraventricular septum thickness but showed slightly smaller correlation coefficient in 1 h (*p* = 0.02. *r* = 0.44 for 1 h, *p* = 0.01, *r* = 0.47 for 3 h). The correlation coefficient between H/CL and left ventricular posterior wall thickness also tended to be smaller at 1 h than at 3 h, and a significant correlation was found only at 3 h but not at 1 h (*p* = 0.12, *r* = 0.30 for 1 h, *p* = 0.04, *r* = 0.39 at 3 h).

**Table 2:** Correlation between H/CL (1 and 3 h) and myocardial wall thickness parameters

	Interventricular septum thickness		LV posterior wall thickness	
	*p*	*r*	*p*	*r*
H/CL 1 h	0.02	0.44	0.12	0.30
H/CL 3 h	0.01	0.47	0.04	0.39

## Discussion

Our study suggests that images obtained 1 h and 3 h after PYP injection may have different utility for the diagnosis of ATTR cardiac amyloidosis, and also indicate limitations of relying solely on the H/CL ratio measured on planar images to diagnose ATTR cardiac amyloidosis.

The present results indicated that H/CL measurements at 1 h may offer less equivocal cases considering wider gap between the highest H/CL of negative cases and the lowest H/CL in 1 h. When planar imaging was performed at 1 h, grade 0, 1 uptake in combination with a H/CL ratio ≤ 1.5 and grades 2, 3 with a H/CL ratio > 1.5 were associated with negative and positive SPECT, respectively. In the study by Castano et al. [5], a H/CL ratio = 1.3 at 3 h was used as the threshold for diagnosis. Recent result from SCAN-MP study also suggested H/CL = 1.3 as recommended threshold at 3 h [13]. However, when we applied this threshold to our data, one patient in the negative group was misdiagnosed as positive based on a H/CL ratio = 1.4 at 3 h (Fig. 5). This case was histologically proven to not be ATTR cardiac amyloidosis. There were two more patients in the negative group without histology who had a H/CL ratio at 3 h > 1.3. The AHA's recommended diagnostic tree uses Perugini grade 2/3 "OR" H/CL ratio exceeding threshold as a positive criterion. It shows that if we follow the diagnostic tree "by the book", the equivocal cases with H/CL ratio on the threshold automatically fall into the positive case. When the overlap between the two groups was too small, the diagnosis of overlapping cases requires additional analysis such as visual assessment, which is susceptible to interobserver variance. Our findings suggest that such a problem should less occur in 1-h images. Imaging at 1 h is advantageous due to patient comfort, fast laboratory throughput, and high-count images allowing the use of a lower radiotracer dose [14].

According to the present results, H/CL value decreases between 1 and 3 h after the injection of the tracer. In the negative cases, it should be due to clearance of PYP from blood pool. In the positive case, it should be due to clearance from both myocardium and blood pool. That should be the reason why the gap between negative case and positive case became larger in 1 h images. On the other hand, the bone uptake of ^99m^Tc-PYP, images taken at 3 h showed higher uptake. This difference in the temporal change between the myocardium and bone, which is concordant with previous study [15], indicates differences in the mechanisms underlying the accumulation of ^99m^Tc-PYP to amyloid deposition in the myocardium and bone. This could elucidate the reason behind the decrease in the H/CL value over time. The ROI placed on the heart and contralateral lung often includes the rib/cartilage area. If the values obtained from the heart and contralateral lung are influenced by bone uptake, the H value becomes H + bone (B) + blood pool (P), and the CL value becomes CL + B. Consequently, the actual calculation for H/CL becomes (H + B + P)/(CL + B). It follows mathematically that as bone uptake (B) increases over time, the H/CL ratio will diminish, gradually approaching 1.

^99m^Tc-PYP is a bone avid tracer that was previously used for myocardial infarct imaging in the 1970s and several binding sites have since been identified in animal experiments [16], including microcalcifications, calcium deposits, and intracellular calcium or intracellular macromolecules. Pepys et al. [17] suggested that a circulating amyloid P component, a universal component of all types of amyloids, binds to amyloid fibrils via a calcium-mediated mechanism, which also explains the uptake of ^99m^Tc-bone avid tracers into amyloid deposits in the myocardium.

We also found correlation between H/CL measured at 3 h and LV wall thickness. It is somewhat concordant with previous findings which showed PYP imaging (as well as other bone tracer imaging) is not only useful in differential diagnosis, but many studies indicate that it is also useful in assessing disease severity and prognosis. There are several studies which indicate bone scan imaging is useful for prognostication [18], monitoring of therapy [19]. Those findings indicate the usefulness of bone scan in ATTR amyloidosis beyond simple differential diagnosis. Our findings parallel the previous findings by Ogasawara regarding myocardial PYP accumulation [20], delineating the progressive nature of amyloid deposition originating from the septal wall and extending across the left ventricular myocardium. The delayed onset of lateral wall PYP accumulation, juxtaposed with the septum, potentially amplifies cardiac dysfunction as amyloid deposition diffusely infiltrates the myocardium. In our findings, the correlation of H/CL with the septum thickness was significant in the 1-h images, but not with the inferior wall. This may also be related to the results of Ogasawara et al. Marume et al. indicated that the magnitude of left ventricular posterior wall thickness clearly correlates with PYP positivity [21]. Interestingly, three of above referenced five studies used 3-h incubation images (and one uses mixed 1-h and 3-h data, one does not clarify the incubation time). In our study, correlation was found only on 3-h data. Considering these factors, PYP imaging proves valuable not only for binary assessments like differential diagnosis, but also for evaluations requiring non-binary measurements, such as assessing disease severity, prognostication, and risk stratification. In this context, a 3-h image may be preferable to a 1-h image.

Many studies indicate the limitation of solely relying on H/CL value measured on planar image for differential diagnosis. Castano et al. compared data from two hospitals using incubation times of 1 and 3 h. When the H/CL value was used as a criterion for differential diagnosis, both sensitivity and specificity were lower for the 3-h incubation than for the 1-h incubation. A study by Pandey et al. analyzed 229 patients enrolled in the SCAN-MP study and found that H/CL = 1.3 on a 3-h image was the recommended threshold, but at the cost of a relatively high false-positive rate (4.2%). The result of our study may concordantly suggest the limitation of planar H/CL value as a diagnostic criterion. It is worth noting that in both the 1-h (H/CL = 1.5) and 3-h (H/CL = 1.3) our datasets, one negative case falls very close to the diagnostic threshold, which indicates a potential for misclassification. As Pandey commented in their article, “optimal combined use of imaging metrics” should be required for accurate diagnosis of ATTR cardiac amyloidosis. In case of equivocal findings on 1-h image, it could be suggested to take SPECT image at 1 and 3 h. Probably, not only in equivocal cases, but in all cases, a SPECT image should be obtained.

There are some limitations that need to be addressed. The most important limitation is the small number of histologically proven cases. In the diagnosis of cardiac amyloidosis, biopsy was previously considered to be necessary to confirm amyloidosis. However, due to the high reliability of ^99m^Tc-PYP (and other bone tracer) imaging and the risks associated with myocardial biopsy, the collection of specimens from the myocardium has recently been avoided. The majority of patients suspected of having cardiac amyloidosis are likely to have heart failure, one of the red flags for cardiac amyloidosis. Therefore, the risks associated with myocardial biopsy are higher than those in normal subjects. In patients with a negative ^99m^Tc-PYP finding, the risks associated with collecting a biopsy sample to confirm the small possibility of cardiac amyloidosis are considered to be high without significant benefits and is the main reason for the low percentage of histologically proven cases. Further studies on a tenfold larger population of histologically proven cases are needed to verify the present results.

## Conclusion

Our study suggests that both 1-h and 3-h incubation times for ^99m^Tc-PYP imaging have different benefits for ATTR cardiac amyloidosis. A one-hour incubation may be preferable for differential diagnostic purposes implicated by the larger gap of H/CL between negative and positive cases, while a three-hour incubation may provide greater utility in evaluating disease severity implicated by the better correlation between H/CL and wall thickness.

## Data Availability

The datasets generated during and/or analyzed during the current study are available from the corresponding author on reasonable request.
